# Galling and Reproduction of Different Isolates of *Meloidogyne floridensis* on Culinary Herbs

**DOI:** 10.2478/jofnem-2025-0006

**Published:** 2025-03-19

**Authors:** Diego A. H. S. Leitão, Ana Karina S. Oliveira, Janete A. Brito, Donald W. Dickson

**Affiliations:** Entomology and Nematology Department, University of Florida, Gainesville, FL 32611, USA; Soil, Water, and Ecosystem Sciences Department, University of Florida, Gainesville, FL 32611, USA. (Current address); Division of Plant Industry, Florida Department of Agriculture and Consumer Services, Gainesville, FL 32608, USA

**Keywords:** *Anethum graveolens*, *Cichorium intybus*, *Foeniculum vulgare*, host status, *Meloidogyne floridensis*, *Nepeta cataria*, *Ocimum basilicum*, *Origanum majorana*, peach root-knot nematode, *Petroselinum crispum*, reproduction factor, *Salvia officinalis*, susceptibility, *Thymus vulgaris*

## Abstract

*Meloidogyne floridensis* was first described in Florida, USA, in 2004 but has since been reported in California, South Carolina, and Georgia. Our objective was to determine the galling and reproduction differences between two isolates of *M. floridensis*, Mf3 and MfGNV14, on culinary herbs. A duplicated study was performed where both isolates were inoculated separately to nine culinary herbs (basil, catnip, chicory, dill, fennel, marjoram, parsley, sage, and thyme). Tomato was used as a susceptible reference. Regardless of the isolate, chicory and marjoram had the lowest gall indices (1.85 and 1.00, respectively) and egg mass indices (1.25 and 0.90, respectively). The reproduction rate of Mf3 was greatest under catnip (959 eggs/g fresh root) and thyme (701 eggs/g fresh root), followed by sage (549 eggs/g fresh root) and parsley (501 eggs/g fresh root). Catnip (2,151 eggs/g fresh root) stood out for number of eggs among all tested herbs, followed by tomato (1,153 eggs/g fresh root) and sage (847 eggs/g fresh root) for MfGNV14. Marjoram was a non-host, chicory, fennel, and thyme were poor hosts, and catnip, parsley, and tomato were good hosts to both *M. floridensis* isolates. Basil, dill, and sage responses were isolate-specific, i.e., resistant to Mf3 but susceptible to MfGNV14.

Agricultural crops are exposed to numerous biotic stressors (e.g., agricultural pathogens) that may suppress their development and hinder their yield potential, impairing food security worldwide. Among such plant pathogens, plant-parasitic nematodes have been associated with over 4,000 plant species (Dayi, 2024), leading to estimated annual global agricultural losses of US$ 80–173 billion (Nicol et al., 2011; Singh et al., 2020). Root-knot nematodes (RKN), *Meloidogyne* spp., are likely responsible for as much as half of such economic losses, ranking first among important soilborne agricultural genera of plant-parasitic nematodes worldwide (Jones et al., 2013).

An emerging species, *M. floridensis*, is now considered an A-rated pathogen in the USA, requiring treatment and eradication practices (Ploeg and Edwards, 2024). The nematode was first described 20 years ago based on morphological, molecular, and cytological analyses, as well as host-range tests, from an isolate collected from roots of the *Meloidogyne-*resistant peach ‘Nemaguard’ (*Prunus persica*) rootstock, Gainesville, FL, USA (Handoo et al., 2004). Later, *M. floridensis* was also found parasitizing other resistant peach rootstocks (i.e., ‘Flordaguard’ and ‘Okinawa’) (Stanley et al., 2009; Nyczepir and Thomas, 2009). Hence, *M. floridensis* is commonly known as the peach RKN. *M. floridensis* distribution is restricted to the USA and is reported in several Florida counties (Brito et al., 2015a). Recently, the species was reported on almond (*Prunus dulcis*) trees grafted onto *M. incognita-*resistant peach rootstocks in California (Westphal et al., 2019), peach ‘Guardian’^™^ rootstocks in South Carolina (Reighard et al., 2019), and several crops in Georgia (Marquez et al., 2020, 2021; Marquez and Hajihassani, 2023).

The increasing number of reports of *M. floridensis* during the past 5 years demonstrates the importance of further evaluating different crop host statuses to improve management practices and decision-making (e.g., crop rotation and use of resistant cultivars). Culinary herbs and spices are valuable cash crops traded globally as commodities (Carlsen et al., 2011). In South Florida, several herbs are cultivated under field conditions, and the tropical and subtropical climate increases these herbs’ vulnerability to RKN infection (Moreno et al., 1992). Although several *Meloidogyne* spp. are known to parasitize culinary herbs, little is known about their parasitism by *M. floridensis*. Our objective was to evaluate the host status of nine of the most consumed culinary herbs to two isolates of *M. floridensis*.

## Materials and Methods

### Meloidogyne floridensis inocula

Both isolates of *M. floridensis* used in this study are part of the RKN collection of the Division of Plant Industry, Florida Department of Agriculture and Consumer Services, Gainesville, FL (Brito et al., 2008). The first isolate, Mf3 (N04-627-53) was originally isolated from cucumber (*Cucumis sativus*) in Hendry County, FL, and the second, MfGNV14, was obtained from a severely galled ‘Flordaguard’ peach rootstock found growing in the University of Florida Fruit Teaching Orchard, University of Florida, Gainesville, Alachua County. The isolates were reared separately on tomato ‘Cobra’ (*Solanum lycopersicum*) in a glasshouse on separate benches to avoid cross-contamination at the University of Florida, Gainesville, FL. Seedlings were inoculated with an egg suspension of 5,000 eggs + second-stage juveniles (J2)/pot for each *M. floridensis* isolate separately. Sixty days after inoculation (DAI), plants were uprooted, and their root systems were thoroughly washed with tap water to remove soil debris and chopped into 2 cm pieces. Egg extraction was performed using a 0.52% NaOCl solution (Hussey and Barker, 1973), as modified by Bonetti and Ferraz (1981). The number of eggs was recorded, and egg suspensions were adjusted to 1,000 eggs/mL of inocula. The inocula were used for the experiments on the same day of extraction.

### Culinary herbs and tomato seedlings

A total of nine culinary herbs were tested: basil (*Ocimum basilicum*), catnip (*Nepeta cataria*), chicory (*Cichorium intybus*), dill (*Anethum graveolens*), fennel (*Foeniculum vulgare*), marjoram (*Origanum majorana*), parsley (*Petroselinum crispum*), sage (*Salvia officinalis*), and thyme (*Thymus vulgaris*). Tomato ‘Cobra’ was included as a known susceptible host. Seeds of all crops were sown into vermiculite in standard plastic seedling trays, three seeds per cell, and placed in a glasshouse for germination. Due to different germination periods, crops were sown on different days to allow for equal development. One week after germination, plants were thinned to one seedling per cell. Four-week-old seedlings of each crop were used in the experiments after thoroughly washing their root systems with tap water to remove vermiculite debris.

### Experiment setup and design

The seedlings were individually transplanted to Ray Leach single cell “cone-tainers” (Stuewe and Sons, Corvallis, OR), hereafter called cones. Since the bottom of the cones contains drainage holes, polyester fiberfill was used in the bottom of each cone to prevent root development out of the system while allowing excess water to percolate. A sandy soil (Hyper-thermic, uncoated Lamellic Quartzipsamments) obtained from a peanut field in Levy County, FL, was heat-pasteurized (88 °C for 1 hour) and passed through a 2-mm pore sieve before being mixed with growing pot media (1:2 v/v). The resulting mixture was used to fill the cones (ca. 150 g/cone) up to 2 cm from the top edges and watered until leakage occurred. Seedlings were transplanted after leaching ceased.

The seedlings (experimental units) were set up in a factorial, completely randomized design, with two levels of *M. floridensis* isolates (Mf3 and MfGNV14), 10 levels of crops (nine culinary herbs and tomato), and five replicates (n = 100) in 2021. Each seedling was inoculated with either Mf3 or MfGNV14 at an initial population (Pi) density of 2,500 eggs + J2/cone/seedling by pipetting 2.5 mL of egg suspension into three small holes surrounding the root system on 22 July 2021. All cones were inoculated within 30 min. After inoculation, the cones were not watered for 24 hours to reduce inocula losses. During the experiment, seedlings were watered whenever the topsoil was completely dry using a squeeze bottle to avoid water-soil splashing, and seedlings were fertilized once a week with Miracle-Gro water-soluble all-purpose plant food (24-8-16) according to the manufacturer’s instructions (The Scotts Company LLC, Marysville, OH).

Each plant’s root system was collected 60 DAI, washed free of soil and/or vermiculite debris, and fresh root weight (FRW) was recorded after excess water was removed with paper towels. Root systems were rated for the presence of root galls (gall index [GI]) and egg masses (egg mass index [EMI]) under a dissecting microscope on a 0 to 5 scale to determine the host reaction as immune, resistant, or susceptible, where 0 = represented no galls or egg masses, 1 = 1 to 2 galls or egg masses, 2 = 3 to 10 galls or egg masses, 3 = 11 to 30 galls or egg masses, 4 = 31 to 100 galls or egg masses, and 5 = >100 galls or egg masses per root system (Taylor and Sasser, 1978). For easier counting of egg masses, root systems were stained with red food coloring (McCormick & Company, Hunt Valley, MD) mixed with distilled water at 12.5% (v/v) (Thies et al., 2002). Egg extraction was performed as mentioned above. The final number of eggs (Pf) for each treatment (*M. floridensis* isolate × crops) was counted under a stereomicroscope SteREO v. 20 (CarlZeiss, Göttingen, Germany) in two replicates using a counting chamber, and the reproduction factor (RF = Pf/Pi) was determined. Due to different crop root system growth, the number of eggs/g fresh root (Eggs/g) was used in statistical analyses. Additionally, the host status of each crop was categorized as good host (GH) when RF ≥ 1, poor host (PH) when 0.1 < RF < 1, or non-host (NH) when RF ≤ 0.1 (Sasser et al., 1984). The experiment was repeated once within the same year (inoculation was performed on 3 October 2021), following the same methodology and evaluating the same dependent variables (EMI, GI, Eggs/g, and RF).

### Statistical analysis

There were no differences between the experiment repeats (unpaired two-sample Wilcoxon test, *P* ≥ 0.05) for EMI, GI, Eggs/g, and RF. Thus, data were pooled across both repeats, and a linear mixed-effect model was used for two-way analysis of variance (ANOVA) to test the main effects and interactions of *M. floridensis* isolates (Mf3 and MfGNV14) and crops (nine culinary herbs and tomato) on the dependent variables. The experiment repeats were considered a random effect. The *lme* function from *nlme* package (Pinheiro and Bates, 2023) was used to create the models using restricted maximum likelihood estimation. Normality and homogeneity of variances were evaluated through inspection of residuals. Whenever ANOVA assumptions were violated, variance function structures (i.e., *varPower* or *varIdent*) were included in the final model to eliminate heteroskedasticity. Marginal means for significant main or interaction effects were estimated using *emmeans* function from the *emmeans* package (Lenth, 2024) and compared using Tukey’s HSD test at a 5% probability level. All analyses were performed on RStudio version 2024.04.2 (Posit team, Boston, MA, 2024).

## Results

**Figure 1:**
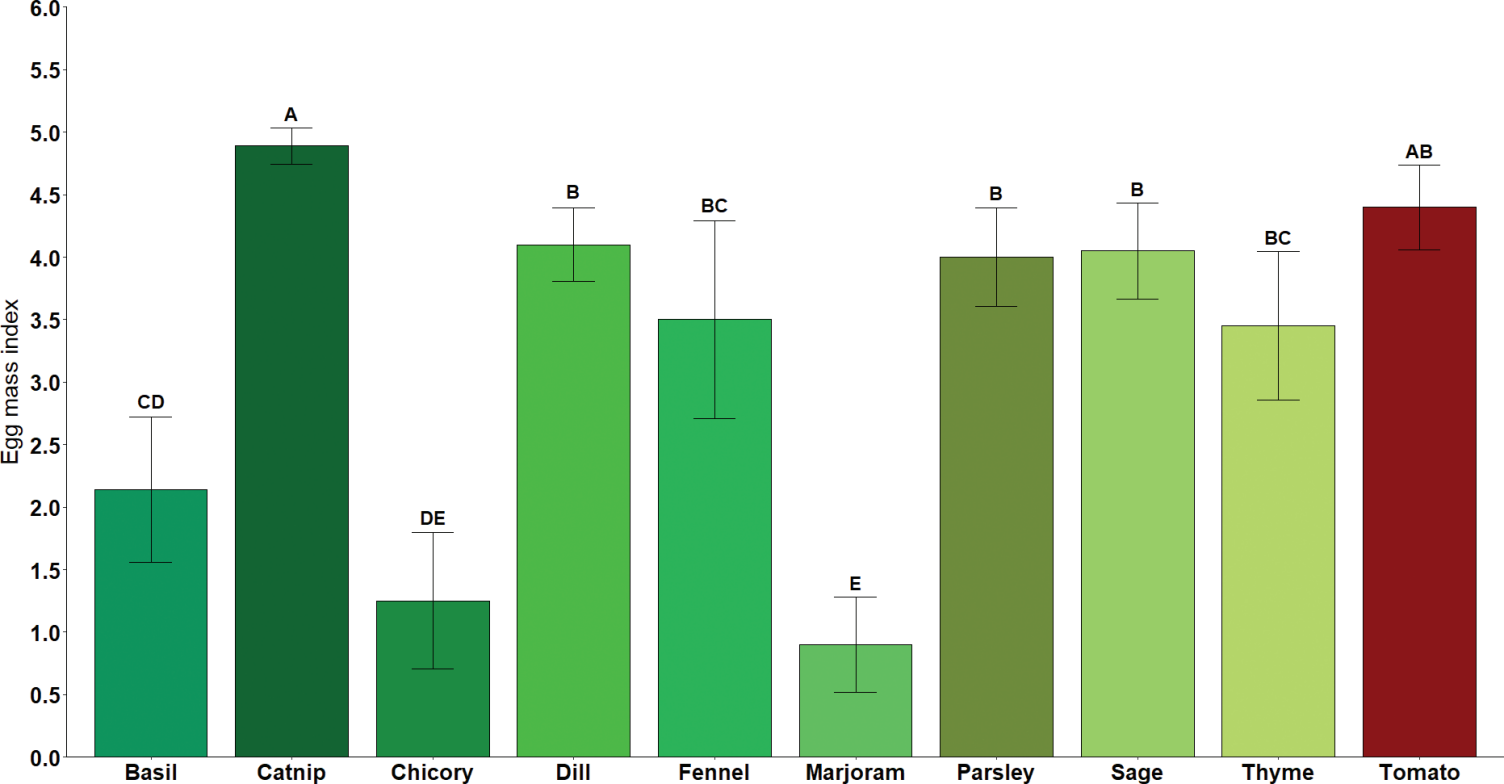
Egg mass index (0 to 5 scale) of different culinary herbs and tomato (susceptible reference) after inoculation with two isolates of *Meloidogyne floridensis*. Bars represent the average of pooled data across isolates and experimental repeats (n = 20), and error bars indicate 95% confidence intervals. Significant differences among culinary herbs are shown by different letters (*P* < 0.05, Tukey’s HSD test). The egg mass index scale is based on the number of egg masses present in the root system (Taylor and Sasser, 1978): 0 = no egg masses, 1 = 1 to 2 egg masses, 2 = 3 to 10 egg masses, 3 = 11 to 30 egg masses, 4 = 31 to 100 egg masses, and 5 = more than 100 egg masses.

Our findings are the first report of *M. floridensis* parasitizing catnip, chicory, fennel, marjoram, and thyme. Differences in average EMI were observed between the two isolates (*P* < 0.05), where MfGNV14 showed higher EMI (3.43) when compared to Mf3 (3.11). The main effect of crops was also significant (*P* < 0.0001), but there was no interaction between factors (*M. floridensis* isolate × plant, *P* ≥ 0.05). Thus, data from Mf3 and MfGNV14 were combined (Fig. 1). The highest average EMI among all culinary herbs was observed for catnip (4.89), which did not differ from tomato (4.40). The dill, fennel, sage, and thyme EMI ranged between 3.45 and 4.10. The lowest EMI was observed for chicory (1.25) and marjoram (0.9) (Fig. 1).

**Figure 2:**
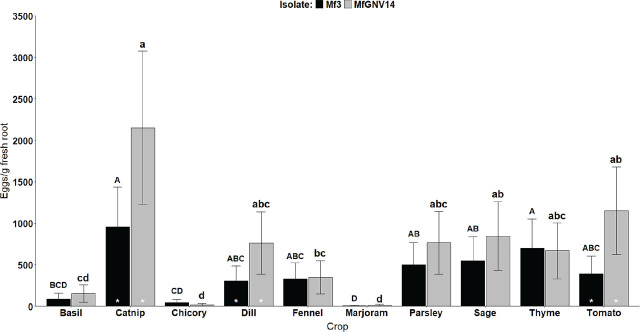
Number of eggs/g fresh root (Eggs/g fresh root) at 60 days from nine culinary herbs and tomato (susceptible reference) after inoculation with two isolates of *Meloidogyne floridensis*, Mf3 and MfGNV14. Bars represent the average of pooled data across experimental repeats (n = 10), and error bars indicate 95% confidence intervals. Different uppercase and lowercase letters determine significant differences among culinary herbs for Mf3 and MfGNV14, respectively (*P* < 0.05, Tukey’s HSD test). The white asterisks at the bottom of the bars indicate differences between *M. floridensis* isolates (Mf3 and MfGNV14) within the same crop (*P* < 0.05, Tukey’s HSD test).

The Eggs/g was affected by *M. floridensis* isolate and plant (*P* < 0.001) alone, as well as their interaction (*P* = 0.01). Thus, data obtained from both experiments were pooled (Fig. 2). For Mf3, the Eggs/g was greatest under catnip (959 eggs/g fresh root) and thyme (701 eggs/g fresh root), followed by sage (549 eggs/g fresh root) and parsley (501 eggs/g fresh root). Similar patterns were observed for MfGNV14. However, catnip (2,151 eggs/g fresh root) stood out among all other crops, followed by tomato (1,153 eggs/g fresh root) and sage (847 eggs/g fresh root). The average number of eggs/g fresh root was lowest for basil (90 and 155 eggs/g fresh root for Mf3 and MfGNV14, respectively), chicory (46 [Mf3] and 18 [MfGNV14] eggs/g fresh root), and marjoram (5 [Mf3] and 13 [MfGNV14] eggs/g of fresh root), which was expected since the least number of egg masses were produced on their root systems, irrespectively of the nematode isolate (Fig. 1). When comparing the level of infection between isolates, MfGNV14 produced comparable number of eggs to Mf3 for basil, chicory, fennel, marjoram, parsley, sage, and thyme. Nevertheless, MfGNV14 produced 2.24, 2.48, and 2.96-fold more eggs/g fresh root than Mf3 in catnip, dill, and tomato, respectively (Fig. 2).

Root galling was observed in 92.5% of all root samples (n = 200), and galling did not depend on the isolate (*P* ≥ 0.05). However, although GI differed among crops (*P* < 0.0001), no interaction with *M. floridensis* isolates was detected; thus, data from both isolates were combined. The data were pooled, and their averages were compared across the culinary herbs and tomatoes (Table 1). Catnip and dill always showed more than 100 galls (i.e., GI = 5). In contrast, basil, chicory, and marjoram showed the lowest average number of galls (i.e., GI = 2.74, 1.85, and 1.00, respectively), differing from all other culinary herbs (i.e., GI > 3) and tomato (GI = 4.85). Only chicory and marjoram resisted Mf3 and MfGNV14 (Table 1).

**Table 1:** Gall index (GI) means (± standard deviation) and the reaction of different culinary herbs and tomato (susceptible reference) 60 days after inoculation with two *Meloidogyne floridensis* isolates, Mf3 and MfGNV14.

**Crop**	**Botanical name**	**GI^a^**			**Reaction**

		**Mf3^b^**	**MfGNV14^b^**	**Average**	
Basil	*Ocimum basilicum*	2.67 ± 1.22	2.80 ± 1.40	2.74C	S
Catnip	*Nepeta cataria*	5.00 ± 0.00	5.00 ± 0.00	5.00A	S
Chicory	*Cichorium intybus*	1.90 ± 1.37	1.80 ± 1.55	1.85CD	R
Dill	*Anethum graveolens*	5.00 ± 0.00	5.00 ± 0.00	5.00A	S
Fennel	*Foeniculum vulgare*	4.40 ± 0.97	3.80 ± 2.10	4.10AB	S
Marjoram	*Origanum majorana*	1.20 ± 0.79	0.80 ± 0.92	1.00D	R
Parsley	*Petroselinum crispum*	4.70 ± 0.48	4.80 ± 0.42	4.75AB	S
Sage	*Salvia officinalis*	4.20 ± 1.03	4.60 ± 0.52	4.40AB	S
Thyme	*Thymus vulgaris*	3.80 ± 0.63	4.10 ± 1.20	3.95B	S
Tomato	*Solanum lycopersicum*	4.80 ± 0.42	4.90 ± 0.32	4.85AB	S

a GI means from duplicate tests were based on a 0–5 scale where 0 = no galls or egg masses, 1 = 1–2, 2 = 3–10, 3 = 11–30, 4 = 31–100, 5 = >100 galls or egg masses per plant (Taylor and Sasser, 1978). The average values (pooled from both isolates and experiments) followed by different uppercase letters are significantly different based on Tukey’s HSD test (*P* ≤ 0.05) and values are to be compared vertically across crops in the same column.

No significant interaction between isolates and crops was observed for GI (ANOVA, *P* > 0.05).

b Mf3 and MfGNV14 were originally isolated from cucumber and peach, respectively.

The reproduction of *M. floridensis* varied by isolate (*P* < 0.01) and plants (*P* < 0.0001), and their interaction was also significant (*P* < 0.05). The RF means were compared among plants for each isolate and between isolates within each plant (Table 2). On average, half of the herbs were good hosts (GH) to MfGNV14 and poor hosts (PH) to Mf3. Furthermore, for Mf3, catnip, parsley, and tomato supported the highest reproduction (RF = 5.65, 1.36, and 1.87, respectively) among all crops. These plants were classified as a GH to Mf3, whereas reproduction was almost absent under marjoram (RF = 0.01), thus being classified as a non-host (NH) to Mf3. All other herbs showed similar reproduction (RF = 0.22–0.85) and were considered poor hosts (PH) to Mf3. Regarding MfGNV14, catnip, dill, parsley, sage, and tomato showed RF ≥ 1. Therefore, these crops are GH to MfGNV14. Like Mf3, marjoram sustained little reproduction of MfGNV14 (RF = 0.02) and was considered an NH for both isolates. The RF between isolates was similar under all plants, except under dill, where MfGNV14 showed a 3.37-fold higher RF than Mf3 (Table 2).

**Table 2:** Reproduction factor (RF) means (± standard deviation) and host status of different culinary herbs and tomato (susceptible reference) 60 days after inoculation with two *Meloidogyne floridensis* isolates, Mf3 and MfGNV14.

**Crop**	**Botanical name**	**RF^a^**		**Host status^c^**	
	
		**Mf3^b^**	**MfGNV14^b^**	**Mf3**	**MfGNV14**
Basil	*Ocimum basilicum*	0.72 ± 0.96 BCa	1.09 ± 1.27 BCDa	PH	GH
Catnip	*Nepeta cataria*	5.65 ± 4.02 Aa	10.20 ± 5.41 Aa	GH	GH
Chicory	*Cichorium intybus*	0.22 ± 0.24 BCa	0.11 ± 0.17 CDa	PH	PH
Dill	*Anethum graveolens*	0.70 ± 0.56 BCb	2.36 ± 1.16 ABa	PH	GH
Fennel	*Foeniculum vulgare*	0.85 ± 0.56 BCa	0.97 ± 0.82 BCDa	PH	PH
Marjoram	*Origanum majorana*	0.01 ± 0.02 Ca	0.02 ± 0.04 Da	NH	NH
Parsley	*Petroselinum crispum*	1.36 ± 1.00 ABa	2.17 ± 2.07 Ba	GH	GH
Sage	*Salvia officinalis*	0.83 ± 1.21 BCa	1.64 ± 1.17 BCa	PH	GH
Thyme	*Thymus vulgaris*	0.32 ± 0.14 BCa	0.72 ± 0.74 BCDa	PH	PH
Tomato	*Solanum lycopersicum*	1.87 ± 2.81 ABa	3.81 ± 3.61 ABa	GH	GH

a RF means from duplicate tests in the same column followed by different uppercase letters are significantly different based on Tukey’s HSD test (*P* ≤ 0.05), and values are to be compared vertically across crops within the same *M. floridensis* isolate. In contrast, RF means in the same row followed by different lowercase letters are significantly different (*P* ≤ 0.05). Values are to be compared horizontally between *M. floridensis* isolates for each corresponding crop.

b Mf3 and MfGNV14 were originally isolated from cucumber and peach, respectively.

c Host status classification was based on the RF values: good host (GH) when RF ≥ 1, poor host (PH) when 0.1 < RF < 1, and non-host (NH) when RF ≤ 0.1 (Sasser et al., 1984).

## Discussion

*Meloidogyne floridensis* was originally considered primarily as a pathogen of peach. However, the number of hosts has increased since its description in 2004 (Handoo et al., 2004), ranging from vegetables and grains to ornamental plants and weeds (Kokalis-Burelle and Nyczepir, 2004; Stanley et al., 2009; Brito et al., 2008, 2010, 2015a, 2015b; Westphal et al., 2019; Marquez et al., 2020). However, little is known about *M. floridensis* infection on culinary herbs, albeit the reaction of herbs to other RKN species has been published previously (Moreno et al., 1992; Walker, 1995; Baida et al., 2011; Brito et al., 2011; Dias-Arieira et al., 2012).

Marjoram was the only culinary herb classified as an NH and resistant to *M. floridensis*, supporting little to no reproduction of both isolates. Unlike other culinary herbs, results on the response of marjoram are more consistent, with most studies reporting this herb as an NH or resistant to different species of RKN (Moreno et al., 1992; Walker, 1995; Baida et al., 2011; Dias-Arieira et al., 2012). There is only one study that reports marjoram as susceptible to *M. enterolobii* (Brito et al., 2011), which is known for its high degree of virulence on many crop plants (Brito et al., 2007a; Oliveira et al., 2020).

Basil and dill were previously classified as GH to *M. floridensis* (RF ≥ 1), whereas parsley and sage showed RF < 1 (Kokalis-Burelle and Nyczepir, 2004). A different cultivar of basil (i.e., ‘Summerlong’) was considered a PH for *M. floridensis* (RF = 0.71) (Mendes and Dickson, 2010). In our study, parsley was a GH for both isolates, with RFs equal to 1.36 and 2.17 for Mf3 and MfGNV14, respectively. Conversely, sage was a PH for Mf3 (RF = 0.83) and a GH for MfGNV14 (RF = 1.64). In addition, basil and dill were PH for Mf3 and GH for MfGNV14. The differences in host status might be related to the isolate virulence and/or the cultivar of the crops used in the experiments. For example, *M. floridensis* isolated from tomato had higher RF in susceptible tomato ‘Talladega’ when compared to an isolate from peach or cucumber (Stanley et al., 2009). Isolates of *M. floridensis* from the same origin (i.e., peach ‘Nemaguard’) showed RF < 1 (Handoo et al., 2004) or RF > 1 (Stanley et al., 2009) under bell pepper ‘California Wonder’. Marquez and Hajihassani (2023) evaluated five different *M. floridensis* isolates and observed that, when the species was isolated from cowpea, it showed a higher level of reproduction than a cucumber-derived isolate on susceptible tomato ‘Rutgers’ (RFs = to 73.2 and 18.7, respectively). However, all five *M. floridensis* isolates showed comparable reproduction (*P* ≥ 0.05, RF between 10.9 and 49.4) under resistant tomato ‘Skyway’. Different host preferences have also been observed in other *Meloidogyne* species, such as *M. enterolobii*, where a Floridian isolate (Brito et al., 2007b) reproduced on *Brassica* spp. (i.e., broccoli and cabbage), whereas a Cuban isolate failed to do so (Rodríguez et al., 2003).

These results underscore the importance of evaluating the reaction of crops to different isolates of RKN since there may be variability in virulence and reproduction levels, which might indicate host races within *M. floridensis* (Stanley et al., 2009). In fact, Handoo et al. (2004) performed a host differential test. They observed that the species had a different host range compared to the two races of *M. incognita*. However, further research needs to be performed using different isolates of *M. floridensis* to ascertain that the peach RKN has different host races. Understanding the reaction of different isolates will be useful for resistance breeding programs, however there is a vast knowledge gap for culinary herbs. Among the evaluated crops, fennel is the only one with documented screening for resistant varieties to *M. incognita* (Kumar et al., 2017) and *M. javanica* (Kumawat et al., 2024).

In summary, our findings showed that *M. floridensis* can parasitize most of the studied culinary herbs, and the present study added catnip as a good host to the list of host plants for the peach RKN. Additionally, marjoram was a nonhost and chicory, fennel, and thyme were poor hosts to both nematode isolates used in this study. Some herbs, therefore, may be used in crop rotation as a management tactic to reduce *M. floridensis* population densities and pressure from other RKNs; however, growers need to be cautious with *M. enterolobii*-infested fields since most culinary herbs have been reported as GH for this nematode species. Our results underscore the potential economic impact of *M. floridensis* on the Floridian and other states’ agricultural industries where this species has been reported. In addition, we suggest future studies to understand the mechanisms of resistance of marjoram to RKN, which in turn might be beneficial for breeding programs since this herb has been consistently an NH for different *Meloidogyne* species.

We observed that *M. floridensis* parasitizes several culinary herbs belonging to different botanical families, namely Lamiaceae (basil, catnip, marjoram, sage, and thyme), Apiaceae (dill, fennel, and parsley), and Asteraceae (chicory), with no evident host status pattern among such families. However, we can highlight differences between *M. floridensis* isolates: six of nine culinary herbs were PH to Mf3, whereas five were GH to MfGNV14. Thus, additional evaluations are needed to determine the pathogenicity among *M. floridensis* isolates.
